# Global isolation by distance despite strong regional phylogeography in a small metazoan

**DOI:** 10.1186/1471-2148-7-225

**Published:** 2007-11-14

**Authors:** Scott Mills, David H Lunt, Africa Gómez

**Affiliations:** 1 Department of Biological Sciences, University of Hull, Hull, HU6 7RX, UK; 2 School of Animal Biology, The University of Western Australia, 35 Stirling Highway, Crawley, WA 6009, Australia

## Abstract

**Background:**

Small vagile eukaryotic organisms, which comprise a large proportion of the Earth's biodiversity, have traditionally been thought to lack the extent of population structuring and geographic speciation observed in larger taxa. Here we investigate the patterns of genetic diversity, amongst populations of the salt lake microscopic metazoan *Brachionus plicatilis s. s*. (sensu stricto) (Rotifera: Monogononta) on a global scale. We examine the phylogenetic relationships of geographic isolates from four continents using a 603 bp fragment of the mitochondrial *COI *gene to investigate patterns of phylogeographic subdivision in this species. In addition we investigate the relationship between genetic and geographic distances on a global scale to try and reconcile the paradox between the high vagility of this species and the previously reported patterns of restricted gene flow, even over local spatial scales.

**Results:**

Analysis of global sequence diversity of *B. plicatilis s. s*. reveals the presence of four allopatric genetic lineages: North American-Far East Asian, Western Mediterranean, Australian, and an Eastern Mediterranean lineage represented by a single isolate. Geographically orientated substructure is also apparent within the three best sampled lineages. Surprisingly, given this strong phylogeographic structure, *B. plicatilis s. s*. shows a significant correlation between geographic and genetic distance on a global scale ('isolation by distance' – IBD).

**Conclusion:**

Despite its cosmopolitan distribution and potential for high gene flow, *B. plicatilis s. s*. is strongly structured at a global scale. IBD patterns have traditionally been interpreted to indicate migration-drift equilibrium, although in this system equilibrium conditions are incompatible with the observed genetic structure. Instead, we suggest the pattern may have arisen through persistent founder effects, acting in a similar fashion to geographic barriers for larger organisms. Our data indicates that geographic speciation, contrary to historical views, is likely to be very important in microorganisms. By presenting compelling evidence for geographic speciation in a small eukaryote we add to the growing body of evidence that is forcing us to rethink our views of global biodiversity.

## Background

Microscopic organisms have long been thought to occur globally wherever suitable ecological conditions exist [1]. Such cosmopolitan distributions are considered to be the consequence of large population sizes and high vagility leading to increased rates of colonization and decreased probabilities of population extinction in comparison to macroscopic organisms [2]. However, recent genetic studies of microbial biogeography in cosmopolitan halophilic bacteria, marine algae and fungi have challenged these views, and indicate that, despite high vagility, microorganisms can be phylogeographically structured [3-6]. Therefore, microscopic organisms may exhibit biological scaling rules similar to those operating on macroscopic organisms; they can be dispersal-limited and have geographic isolation leading to local adaptation, and allopatric divergence [7,8].

Continental zooplanktonic species, including copepods, cladocerans and rotifers, are small, microscopic organisms that disperse passively through diapausing propagules and are often found in large densities in ponds and lakes. In consequence, they were thought to follow microbial rules of biogeography often summarized in the phrase "*Everything is everywhere, but, the environment selects*[9]." Despite being a life-long proponent of the dominance of allopatric speciation Ernst Mayr, one of the architects of the evolutionary synthesis, wrote:

"Probability of passive dispersal is increased by numerous factors [...] such as small size, low specific gravity, protective coating, a dormant stage, and so forth. Species that are optimal for all these factors may have world-wide ranges, such as certain tardigrades, rotifers, and fresh-water crustaceans. A successful cosmopolitan with an essentially panmictic species population is evidently barred from geographic speciation [10]." This perception has persisted almost unchanged until this century [11].

In striking contrast to these views, a paradigm shift seems to be occurring, as illustrated by a number of recent phylogeographic and population structure analyses. These studies uncover high levels of genetic structuring at a regional scale amongst zooplankton populations, and indicate very low levels of gene flow despite the high potential for these organisms to disperse [12-17].

In this study, we investigate the patterns of genetic diversity found amongst populations of the euryhaline microscopic metazoan *Brachionus plicatilis s. s*. (sensu stricto) (Rotifera: Monogononta) on a global scale using a fragment of the cytochrome *c *oxidase subunit I gene (*COI* or *cox1*) gene. We try to reconcile the seemingly contradictory high vagility of this species with the observed patterns of restricted gene flow, even over local spatial scales. Our analysis of new and published data on mitochondrial DNA sequence variation underscores the strong phylogeographic structure of *B. plicatilis s. s*. both between and within continents, indicating the operation of incipient allopatric speciation. In addition, and surprisingly, given the non-equilibrium conditions and strong genetic drift operating in the system, a strong correlation between genetic and geographic distance is found. This paradoxical situation suggests that the colonization process by itself can create such patterns. Our results indicate that the evolutionary processes operating in small metazoans are similar to those known to be occurring in macro-organisms. Our observations challenge the intuitive dogma that high vagility leads to decreased biodiversity. As small eukaryotes constitute a remarkably diverse group, being amongst the most numerous organisms on the planet [18], the understanding of phylogeographical subdivision in such taxa has profound consequences for the formulation and interpretation of global patterns of biodiversity.

## Results

### Patterns of genetic variation and phylogenetic relationships

**Figure 1:**
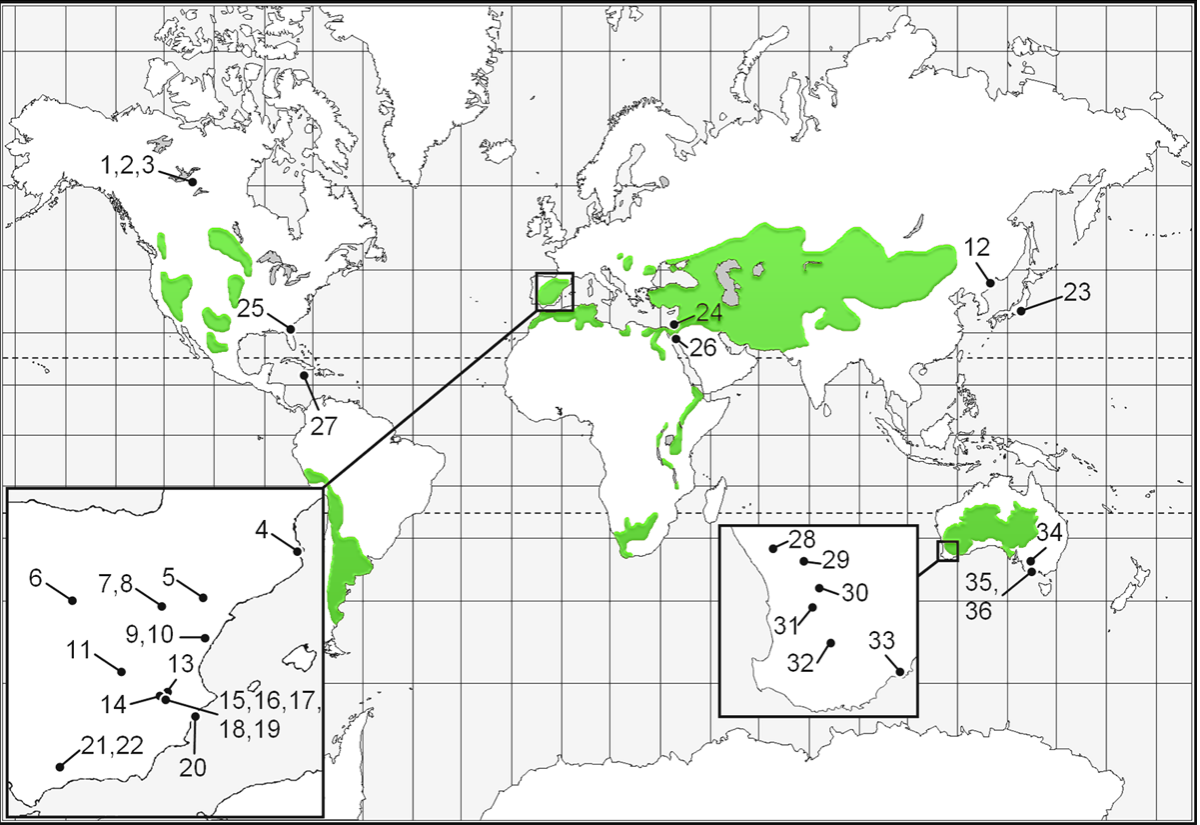
**Global distribution of the 36 sites containing *Brachionus plicatilis s. s*. populations used in the current study**. Areas shaded green represent the major endorheic basins of the world [36]. See Table 1 for more detailed site information.

Of the 36 Australian lakes sampled, 22 contained diapausing eggs belonging to the *B. plicatilis *species complex. A total of 353 diapausing eggs were recovered and processed for DNA extraction and PCR amplification of the COI fragment with 8 sites yielding 13 new *B. plicatilis s. s*. sequences. To these, we added 30 additional sites from previous studies, represented by 135 sequences [16,19-21]. Given that some of the individuals previously sequenced came from aquaculture facilities the geographic origin of two sequences could not be accurately established, these sequences were removed from the dataset (Nhi1; [GenBank: AY785182] and Amami; [GenBank: AY785174], [19]). The complete dataset represents 36 sites from around the world (Figure 1), with the 603 base pair alignment collapsed to 52 haplotypes.

In total, the alignment contained 120 polymorphic sites, 89 of which were parsimony informative. The entire data set displayed a high level of synonymous substitution with only six polymorphic sites resulting in amino acid substitutions in individuals from Hay Camp [GenBank: AF499054], Laguna de las Eras [GenBank: AF266895], Torreblanca Marsh Poza Norte [GenBank: AF266858], Forgotten Lake [GenBank: AF499055, GenBank:AF499056], all Western Australian sequences [GenBank: EF524543-EF524555] and Grosbeak Lake [GenBank: AF499057-AF499063, GenBank: AF499066-AF499069].

The model of nucleotide substitution that best fits the data is the transition model with unequal base frequencies and rate variations among sites following a gamma distribution (TIM + G shape parameter 0.18). Raw sequence divergence (p-distance) ranged from 0.17% to 8.29% (0.16% to 11.91%, ML patristic distances).

**Figure 2:**
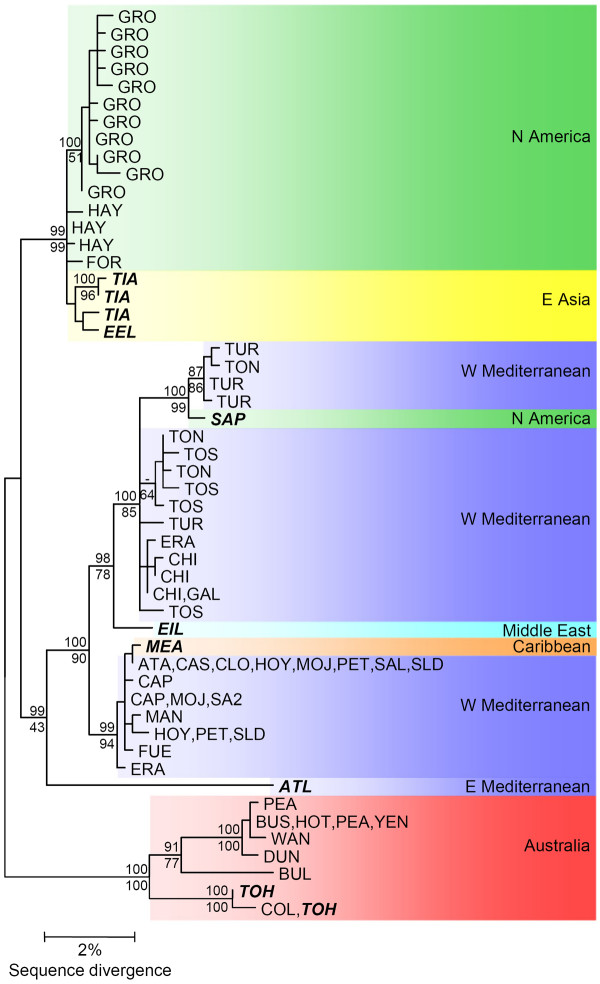
**A midpoint rooted ML phylogenetic tree for *B. plicatilis s. s*. based on *COI *sequences**. Identical sequences were collapsed by haplotype and are indicated by a sample site code as indicated in Table 1. Values above branches represent posterior probabilities for nodes from the Bayesian analysis while those below branches indicate ML bootstrap support (1000 pseudoreplicates). Names in bold italics represent laboratory cultures.

The inferred phylogenetic relationship of *B. plicatilis s. s*. haplotypes is shown in Figure 2. Both ML and Bayesian phylogenetic methods retrieved the same gross topology with well-supported main branches differing only in minor rearrangements of the leaves (involving sequences from Grosbeak Lake and Torreblanca Marsh). A strong geographical orientation to the tree topology is evident, with four geographically distinct clades (Figure 2). A first clade was formed by all Australian sequences, a second one containing North American and Far East Asian sequences, a third one with an Eastern Mediterranean sequence, and a fourth one with mostly Western Mediterranean sequences. Three exceptions to this geographic patterning are apparent in the Western Mediterranean clade. The first exception from the Caribbean (MEA; [GenBank: AY785189]) differed by a unique single base substitution from sequences found in several lakes on the Iberian Peninsula (ATA, CAS, CLO, HOY, PET, SAL and SLD; [GenBank: AF266929-AF266950]), while the second exception from the East coast in the USA (SAP; [GenBank: AY785187]) differed by four synonymous substitutions from a Spanish haplotype (TUR; [GenBank: AF266853-AF266855]), two of these substitutions were unique to the Sapelo sequences. In both cases individuals closely related to European populations are found in Atlantic American locations. A third sequence (EIL; [GenBank: AY785188]) falls within this group although it was sampled from the coast of the Red Sea. A pattern of geographic substructure is apparent within some of these lineages. The Australian clade contains Eastern and Western groups and a strong substructure in the Western Mediterranean lineage is well illustrated within the Iberian Peninsula.

### Analysis of association between genetic and geographic distances

**Figure 3:**
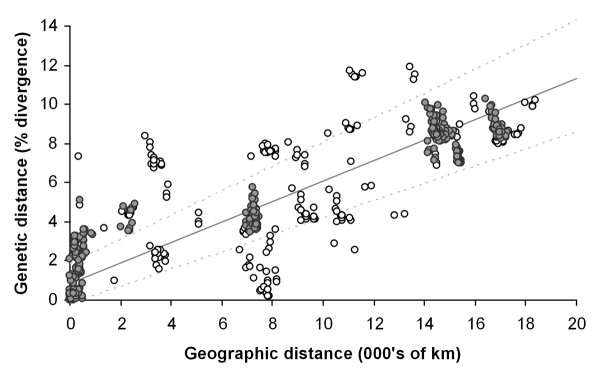
**Graph of RMA regression for pairwise geographicdistances and *COI *TIM + G corrected genetic distances**. The RMA 95% confidence intervals for the slope and intercept, as estimated from 100,000 bootstraps, are plotted in combination as broken lines. Pairwise comparisons involving sites represented by laboratory kept clones (ATL, ELI, EEL, MEA, SAP, TIA and TOH) have been marked with open circles.

Figure 3 shows a scatterplot of the pairwise corrected genetic distances (TIM + G) versus great circle geographic distances. Measures of geographic and genetic distance exhibited a highly significant positive correlation in the RMA analysis indicating that geographic distance explains a large proportion of the variance in genetic distance between populations (*r*^2 ^= 0.73, *P *= < 0.01, *y *= 1.11 × 10^-3 ^+ 5.25 × 10^-6^*x*).

**Figure 4:**
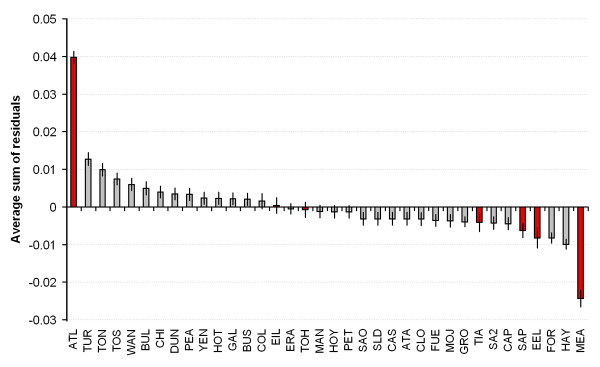
**Average sum of residuals by sample site for the RMA regression analysis**. Laboratory kept clones are shown in red and error bars indicate the standard error of the mean. Site abbreviations as in Table 1.

Consideration of the regression residuals revealed that the largest deviation from the regression model was represented by pairwise comparisons involving two laboratory kept clones (sites ATL and MEA see Figure 4 and Table 1). In the case of ATL, a larger than expected genetic distance was found, in the case of MEA a smaller than expected genetic distance was found. Although natural events could be responsible for this pattern, laboratory clones are exposed to contamination and mislabelling problems. Removal of all laboratory kept clones from the RMA analysis (representing 5 localities) markedly increased the correlation coefficient between geographic and genetic distance (*r*^2 ^= 0.91, *P *= < 0.01, *y *= 1.13 × 10^-2 ^+ 4.93 × 10^-6^*x*).

**Table 1:** Details of field sites where *Brachionus plicatilis s. s*. individuals used in this study were sourced

**#**	**Code**	**Site Name**	**Country**	**N**	**Latitude**	**Longitude**
1	GRO	Grosbeak Lake	Canada	11	59.792	-111.996
2	FOR	Forgotten Pass Lake	Canada	2	59.58	-111.451
3	HAY	Hay Camp Lake	Canada	3	59.541	-111.465
4	TUR	Estany d'en Turies	Spain	7	42.243	3.103
5	CHI	Salada de Chiprana	Spain	8	41.239	-0.182
6	ERA	Laguna de las Eras	Spain	10	41.202	-4.582
7	SA2	Balsa de Santed II	Spain	10	41.016	-1.542
8	GAL	Laguna de Gallocanta	Spain	5	40.974	-1.508
9	TON	Torreblanca Marsh, Poza Norte	Spain	4	40.148	0.17
10	TOS	Torreblanca Marsh, Poza Sur	Spain	12	40.146	0.168
11	MAN	Laguna de Manjavacas	Spain	9	39.416	-2.866
12	***TIA***	Tianjin	China	3	39.158	117.176
13	SAL	Laguna del Salobrejo	Spain	5	38.914	-1.469
14	PET	Laguna de Pétrola	Spain	9	38.84	-1.566
15	MOJ	Laguna de Mojón Blanco	Spain	2	38.798	-1.432
16	SLD	Laguna del Saladar	Spain	4	38.789	-1.419
17	HOY	Laguna de Hoya Rasa	Spain	2	38.785	-1.428
18	CAS	Laguna de Casa Nueva	Spain	2	38.776	-1.433
19	ATA	Laguna de Atalaya de los Ojicos	Spain	3	38.773	-1.43
20	CLO	Clot de Galvany	Spain	2	38.246	-0.546
21	FUE	Laguna de Fuente de Piedra	Spain	2	37.109	-4.767
22	CAP	Laguna de Capacete	Spain	5	37.016	-4.842
23	***EEL***†	Eel Pond	Japan	4	34.717	136.533
24	***ATL***	Atlit coast	Israel	1	32.703	34.94
25	***SAP***	Sapelo	USA	1	31.5	-81.219
26	***EIL***	Eilat	Israel	1	29.56	34.967
27	***MEA***	Meagher Pond	USA (Caribbean)	1	19.295	-81.232
28	WAN	Lake Wannamal	Australia	2	-31.109	116.049
29	PEA	Pearse's Lake	Australia	3	-31.629	116.914
30	YEN	Yenyenning Lakes	Australia	1	-32.235	117.233
31	HOT	Hotham River	Australia	1	-32.64	116.977
32	BUS	Bushy Swamp	Australia	2	-33.541	117.27
33	DUN	Dunn's Swamp	Australia	1	-33.924	120.155
34	BUL	Lake Bulla	Australia	1	-34.755	142.361
35	COL	Lake Colongulac	Australia	2	-38.173	143.163
36	***TOH***	Tower Hill Lake	Australia	5	-38.325	142.368

# = Code used in Figure 1. Code = Lake code used in Figure 2 and text. N = Number of sequences. All coordinates are given in decimal format with southern latitudes and eastern longitudes represented by negative values. Codes in bold italics represent laboratory cultures. † = Only the approximate location of Eel Pond is known.

## Discussion

Analysis of *B. plicatilis s. s*. mitochondrial sequences from populations in eight countries reveals a strong positive correlation between genetic and geographic distances in this passively dispersed species. Such a pattern, usually referred to as 'isolation by distance', is typically interpreted as indicative of migration-drift equilibrium [22], and might even be expected given the traditional view that large population sizes and high dispersal rates preclude geographic speciation in small cosmopolitan species [2,8,9,23]. However, it does not seem likely that this pattern is due to the global species having attained migration-drift equilibrium, as strong population genetic differentiation even between nearby lakes has been demonstrated in this species [16,17], and here we show extensive structuring of continental populations into geographically restricted phylogenetic lineages. While there is strong evidence for efficient dispersal capacity of small organisms [24], there is a growing realisation that continental and global zooplankton populations are far from migration-drift equilibrium. Allozyme and microsatellite markers have shown that population subdivision is often very strong in cladocerans, rotifers and anostracan populations, indicating a large contribution of genetic drift, and little effect of gene flow in population structure [13,14,16,24-26]. Following Wright's island model [27], migration-drift equilibrium would be extremely slow to attain in organisms with large population sizes after a founding event [12]. This is especially true of rotifers in the *B. plicatilis *species complex where large resting egg banks (2.9 – 13.6 × 10^4 ^eggs per m^-2^) [28] and high densities of adults in the water column (≈ 1.7 × 10^4 ^individuals.L^-1^)[29] are observed in natural populations. Given their rapid reproductive rates and short generation times newly colonized suitable habitats may be rapidly filled following the arrival of a single propagule.

Instead of a migration-drift equilibrium scenario being responsible for the 'isolation-by-distance' patterns we suggest instead that, in a similar way to humans [30], such patterns can be generated not only in equilibrium conditions, but through colonization patterns and drift when colonization is from nearby populations. In this alternative scenario, dispersal vectors work more successfully across small distances with newly formed habitats being more often colonized from local sources. In addition resident population numbers and diapausing egg banks act as an effective buffer to dispersal by way of intraspecific competitive exclusion, indicating that established populations are the major barrier to dispersal. This barrier to dispersal has been described as the Monopolization Hypothesis by De Meester *et al*. [24] who also indicated that local adaptation may enhance the buffering capacity of a resident population against migration.

Interestingly, populations exhibiting positive or negative residuals in the RMA regression may represent either similar or quite divergent scenarios. Those populations that display negative residuals, as their pairwise comparisons indicate a larger than expected geographic distance for their genetic divergence, are likely to be the result of long-distance migration or belong to populations that have experienced range expansions. Such a situation is exemplified by the sequences from Sapelo (SAP; [GenBank: AY785187]) and especially Grand Cayman Island (MEA; [GenBank: AY785189]) that appear to have originated from Spain or North Africa. Conversely, populations having positive residuals, including mainly pairwise comparisons involving Spanish populations, but also ATL in the Eastern Mediterranean, exhibit greater than expected genetic distances for their geographic distance. These samples may represent a situation whereby regional populations have started to undergo geographic and reproductive separation. Alternatively, historical habitat fragmentation with recent recolonisation from refugia may account for these outliers, potentially located near the contact zones between divergent lineages. This inference is supported by Gómez *et al*. [16] who suggested that early Pleistocene fragmentation of *B. plicatilis s. s*. populations was characterized by reduced gene flow and occasional long distance dispersal in the Iberian Peninsula, a pattern that would be consistent with post glacial colonization of this area from two refugia, perhaps one in Spain itself and the other in Northern Africa.

Consideration of the regression residuals revealed that a large proportion of deviation from the regression model was accounted for by some laboratory kept clones (Figures 3 and 4). It cannot be discounted that some of the original clones may have been cross contaminated or mislabelled during many years of maintenance in the laboratory. However, even if this might have been the case, the pattern we found remains strong, and the possible sources of error do not detract from a robust pattern. For this reasons even though "wild" populations of microscopic metazoans should be favoured over those from laboratory sources in future research, the information added by laboratory populations remains highly valuable.

A consequence of dispersal barriers, whether due to habitat monopolization or other processes, is geographic subdivision likely leading to allopatric speciation. Although largely excluded as an important process in small cosmopolitan organisms until recently, it is becoming clear that microscopic and macroscopic organisms may not have such radically different responses to evolutionary forces. Although population genetic divergence can be a good indicator of reproductive isolation [31], such correlations can rarely be included in phylogeographic studies due to the difficulty of their measurement. Reproductive isolation experiments in *B. plicatilis s. s*. have shown a steady and significant decline of fertilization success at relatively low genetic distances of less than 8% *COI *divergence (Suatoni *et al*. unpublished manuscript). In light of this decay of reproductive compatibility, the pattern of genetic divergence we observe on a global scale has evolutionary significance, most likely indicating incipient geographic speciation. This is especially true when we consider that the most distantly separated populations of *B. plicatilis s. s*. exhibit a *COI *p-distance of 8.29%, which approaches the level of divergence between species in the *B. plicatilis *species complex [21].

*Brachionus plicatilis s. s*. global populations are divided into a minimum of four mitochondrial lineages that are geographically structured in a nearly continental fashion: Australian, Western Mediterranean, Eastern Mediterranean and North America-Far East. We are aware that large geographic regions containing salt lakes have not been sampled for this species, in particular Sub-Saharan Africa and Central Asia. Sampling of other areas is sparse, so the diversity we have uncovered is likely to be a subset of that in *B. plicatilis s. s*. An isolated sample from the Eastern Mediterranean (Atlit, Israel) forms a fourth lineage, which appears as the basal branch to the Western Mediterranean group and this could possibly represent the distribution limits of otherwise unsampled African or Asian lineages.

## Conclusion

We have demonstrated a strong global signal of IBD in a passively dispersing species with a discontinuous patchy habitat, very large population sizes, and pronounced local genetic structuring. We conclude however that this does not indicate that migration drift equilibrium has been attained, but rather that it is likely to have been produced through the colonization process. Contrary to the failing paradigm prevalent in the literature since Darwin's time [32], even potentially dispersive microscopic cosmopolitan organisms can achieve substantial geographic subdivision. This subdivision can commonly lead to reproductive incompatibility. Although the number of cosmopolitan micro-species examined for phylogeographic structure is still very small, we suggest that it would be very unwise to assume a priori that radically different evolutionary processes are operating on their phylogeography than those on macro-organisms. Furthermore, geographic components of intraspecific diversity need to be more closely considered in future studies involving microorganisms, especially when considering levels of global biodiversity. This is likely to manifest in the recognition of cryptic species complexes within taxa traditionally thought to be cosmopolitan.

## Methods

### Study species

*Brachionus plicatilis s. s*. is part of a relatively well-known species complex that is composed of more than a dozen species as elucidated by morphological, phylogenetic and reproductive isolation experiments [19,21,33]. *Brachionus plicatilis s. s*. is a cyclical parthenogen with sexual reproduction giving rise to dormant diapausing eggs (90–150 μm long). As in other zooplankton, these eggs are resilient to desiccation and environmental insults [28,34] and may be passively transported for long distances [35]. *B. plicatilis s. s*. has been found inhabiting estuaries, lagoons, rivers and saline lakes around the world with no records in the open ocean. The primary habitats for the species complex are salt lakes that have no connection with a sea or ocean, occurring in endorheic basins [36] (Figure 1). Such lakes are often shallow and subject to considerable environmental fluctuations across several temporal scales, ranging from near fresh through hyper-saline, and from permanent through seasonal, thus representing a mosaic of extreme island-like habitats.

### Sample collection and diapausing egg isolation

To add to the database of *B. plicatilis s.s*. mitochondrial *COI *sequences already available, particularly from North America and Western Europe, we collected soil sediments from 36 saline or brackish water bodies from the Australian continent between January 2002 and May 2004. Sampling focused on south west Australia, with several additional sites from eastern Australia. The south west Australian sites had been previously indicated by the Department of Conservation and Land Management (CALM) as containing members of the *B. plicatilis *species complex [37] and thus were chosen as good candidates for the retrieval of diapausing eggs.

Sampling diapausing egg banks has been acknowledged as a cost-effective and efficient technique for assessing a site's total diversity, especially in fluctuating environments where closely related species can be involved in seasonal succession and preferential hatching mediated by habitat state can lead to sampling bias [28,38-41]. To maximize the chance of obtaining a representative collection of the sampled lakes' diapausing egg bank, and hence genetic diversity, samples from the superficial sediment, to a depth of approximately 2 cm, were collected at various pooling points within each lake.

Diapausing eggs were recovered with a sugar flotation method [42] modified from Gómez and Carvalho [38]. Four replicate samples of sediment, each with an approximate sediment volume of 25 cc, were suspended in 40 mL of 1.75 molar sucrose solution in a 50 mL tube. These samples were then centrifuged at 100 G for 5 minutes. The resulting supernatant was poured through a 45 μm pore-size nylon mesh, and washed with distilled water. Residual particulate matter was washed into a Petri dish containing distilled water where individual diapausing eggs were located and collected using a 10 μL pipette tip under a dissecting microscope.

### DNA extraction, PCR amplification and sequencing

In preparation for DNA extraction, diapausing eggs were washed three times in distilled water, by way of serial transfer between Petri dishes, placed in individual sterile 200 μL centrifuge tubes containing 35 μL of InstaGene Matrix [Bio-Rad Laboratories] and had their contents released by crushing them against the side of the tube. The samples were then heated to 56°C for 20 min, 100°C for 10 min, and then cooled at 4°C for 30 min. All DNA extractions were stored at 4°C until needed.

A 712 bp fragment of the *COI *gene was amplified by PCR with LCO1490 and HCO2198 primers [43] for each individual sample. Reactions were prepared in a 10 μL volume containing 1× PCR Buffer, 1.5 mM MgCl_2_, 200 μM dNTPs, 2.5 pMol or both *COI *primers, 0.125 units of *Taq *(Bioline) polymerase, and 2 μL of the supernatant of the DNA extraction. Samples were then subjected to the following thermocycling profile: 1 cycle of 3 minutes at 93°C, 40 cycles of 15 seconds at 92°C, 20 seconds at 50°C, and 1 minute at 70°C, with a final incubation of 3 minutes at 72°C.

PCR products were sequenced on a CEQ 8000 Genetic Analysis System (Beckman Coulter Laboratories) using CEQ DTCS (Dye Terminator Cycle Sequencing) Quick Start Kits (Beckman Coulter Laboratories) and CEQ LPA-1 (linear polyacrylamide) denaturing gel, following ethanol precipitation and cleanup. Complementary strands of DNA were sequenced and all inconsistencies and polymorphisms were manually checked against original chromatograms in CodonCode Aligner v 1.3.4 (CodonCode Corporation). All *B. plicatilis s. s. COI *sequences were deposited in GenBank's International Sequence Databases [GenBank: EF524543-EF524555].

In addition *B. plicatilis s. s. COI *sequences from previous studies [16,19-21] were used in the analysis [GenBank: AF266853-AF266883, AF266885-AF266950, AF387244-AF387245, AF499054-AF499069, AY785175-AY785181 and AY785183-AY785193].

### Phylogenetic reconstruction

We carried out a maximum likelihood (ML) phylogenetic reconstruction implemented in PHYML [44], using the model of nucleotide substitution estimated from log-likelihood parameters following the Akaike information criterion in ModelGenerator [45]. Support for the topology was tested using 1000 bootstrap pseudoreplicates. In addition, a Bayesian analysis was conducted to obtain a phylogenetic reconstruction with posterior probabilities for nodes using 2,000,000 Markov chain Monte Carlo generations in MrBayes 3.1.1 [46]. Summaries of this analysis were derived with a burnin value determined by the asymptotic convergence of two independent runs as noted by the average standard deviation of the split frequencies sampled every 100 generations.

### Analysis of association between genetic and geographic distances

Raw *COI *p-distances were calculated in PAUP* [47], while patristic distances were taken from the tree derived from the PHYML ML analysis using PATRISTIC [48]. For lakes containing more than one haplotype, averages of pairwise genetic distances were calculated with equal weighting given to haplotypes as sampling bias is likely to have been high due to sites being represented by only a few individuals.

Distances between field sites were estimated using the great arc formulae for calculating distances on a spheroid, with a value of 6371.01 km as the mean radius of the Earth [49]. For all sequences of known geographic origin decimal longitudes and latitudes were recorded with the aid of virtual Globe [50]. The distance between locations in kilometers was calculated with the following formula:

Distance = 6371.01 × ACOS [COS[RADIANS[90-lat1]] × COS [RADIANS[90-lat2]] + SIN [RADIANS[90-lat1]] × SIN [RADIANS[90-lat2]] × COS [RADIANS[long1-long2]]]

Where lat1 and lat2 are the latitudes of two given sites, 1 and 2, and long1 and long2 are the longitudes of these two sites. In order for the equation to function correctly, southern latitudes and eastern longitudes were entered as negative decimal values while positive decimal values were assigned to northern latitudes and western longitudes. The error of great circle calculations is <1% of the given estimate for the entire globe across all scales.

Reduced major axis (RMA) linear regression between genetic and geographic distances was performed on corrected *COI *distances, TIM + G as selected by ModelGenerator [45], for the entire dataset. As the regression contained multiple pairwise comparisons the Mantel test for significance was employed with 100,000 randomizations. The 99% Confidence intervals were also estimated with 100,000 bootstraps over independent population pairs. All regression related analyses were carried out in IBD v1.52 [51].

## Authors' contributions

SM carried out the molecular genetic studies, contacted researchers to profile geographic data and carried out the phylogenetic analysis. SM, DHL and AG conceived and designed the study and jointly wrote the manuscript. All authors read and approved the final manuscript.
